# Chinese Herbal Formula Xuefu Zhuyu for Stable Angina (CheruSA): Study Protocol for a Multicenter Randomized Controlled Trial

**DOI:** 10.1155/2020/7612721

**Published:** 2020-08-31

**Authors:** Shaojun Liao, Zhe Zhang, Geng Li, Li Zhou, Junwen Jiang, Ni Zhang, Yang Wang, Yi Du, Zehuai Wen

**Affiliations:** ^1^Second Clinical Medical College (Second Affiliated Hospital), Guangzhou University of Chinese Medicine, Guangzhou 510405, China; ^2^Liaoning University of Traditional Chinese Medicine, Shenyang 110847, China; ^3^Key Unit of Methodology in Clinical Research, Guangdong Provincial Hospital of Chinese Medicine, Guangzhou 510120, China; ^4^Mathematical Engineering Academy of Chinese Medicine, Guangzhou University of Chinese Medicine, Guangzhou 510405, China; ^5^Cardiovascular Department, Affiliated Hospital of Liaoning University of Traditional Chinese Medicine, Shenyang 110032, China; ^6^State Key Laboratory of Dampness Syndrome of Chinese Medicine, Second Affiliated Hospital of Guangzhou University of Chinese Medicine, Guangzhou 510120, China

## Abstract

*Introduction.* Stable angina (SA) in coronary heart disease is a common ischemic heart disease endangering the patient's quality of life and longevity. Clinical trials have demonstrated that the Chinese herbal formula Xuefu Zhuyu (XFZY) has benefits for SA patients. However, there remains a lack of high-quality evidence to support clinical decision-making. Therefore, we designed a randomized controlled trial (RCT) to evaluate the efficacy and safety of XFZY for SA. *Methods and Analysis.* This multicenter, double-blinded RCT will be conducted in China. 152 eligible participants will be randomly assigned to either an XFZY group or a control group at a 1 : 1 ratio. Participants in the XFZY group will receive XFZY plus routine care, while those in the control group will receive placebo plus routine care. The study period is 26 weeks, including a 2-week run-in period, a 12-week treatment period, and a 12-week follow-up. The primary outcome is the change in visual analogue scale score for angina pain intensity from baseline to 12 weeks. The secondary outcomes are the angina attack frequency and duration, the nitroglycerin dosage consumed, the Canadian Cardiovascular Society grading of effort angina, the Seattle Angina Questionnaire, the EuroQol-5-Dimensions-5-Level, the incidence of major adverse cardiac events, health cost evaluation, and overall assessment for study drugs. *Ethics and Dissemination.* The study has been approved by the ethics committee of Guangdong Provincial Hospital of Chinese Medicine (approval no. BF2019-175-01). Results will be submitted for publication in peer-reviewed journals and disseminated at scientific conferences. This trial is registered with ChiCTR1900026899, registered on 26 October 2019.

## 1. Introduction

Stable angina (SA) is a common condition among coronary heart disease (CHD) patients. It is characterized by pain in the substernal chest or adjacent areas caused by myocardial ischemia [1]. According to a survey from 2011 to 2014, the prevalence of angina pectoris among adults is 3.4% in the United States [2]. In China, the prevalence of angina is up to 9.4% [3]. Patients with SA are at higher risks of cardiovascular events and sudden cardiac death, with an annual mortality rate of up to 3.2% [4]. Therefore, SA management is of paramount importance. However, available anti-ischemic agents are limited due to their negative hemodynamic and electrophysiologic effects [5, 6]. It has been clinically reported that percutaneous revascularization relieves symptoms better than antianginal medication, but this advantage diminishes over time [7]. And in fact, percutaneous coronary intervention (PCI) did not increase exercise time or relief symptoms more than a placebo procedure among patients with medically treated angina and severe coronary stenosis [8]. Many patients who are on treatment for SA remain symptomatic. Nearly 40% of them declare that this illness impacts their quality of life completely and 10% rate their condition as “not good” [9]. Chinese medicine (CM), which has a long history in China and other Asian countries, could provide further options for SA. In China, a large majority of SA patients seek help from CM, in addition to conventional treatments [10]. Moreover, clinical trials have revealed that CM and ingredients derived from Chinese herbs benefit patients with SA [11].

Xuefu Zhuyu decoction (XFZY), a classical Chinese herbal formula, is the representative Chinese herbal medicine prescription for the treatment of SA. It is prevalent in the treatment of SA with *qi-stagnation and blood-stasis pattern* (*QBP*) (*Zheng* or syndrome in CM) [12]. XFZY is a patented product that comes in various forms that are included in the 2015 edition of the Chinese Pharmacopoeia. It was approved by the China Food and Drug Administration in 2002 (Approval no. Z10950063) and has been used extensively for over 15 years [13, 14]. XFZY is composed of *Persicae Semen* (Taoren), *Carthami Flos* (Honghua), *Rehmanniae Radix* (Dihuang), *Angelicae Sinensis Radix* (Danggui), *Chuanxiong Rhizome* (Chuanxiong), *Paeoniaeradix Rubra* (Chishao), *Achyranthis Bidentatae Radix* (Niuxi), *Platycodonis Radix* (Jiegeng), *Bupleuri Radix* (Chaihu), *Aurantii Fructus* (Zhiqiao) fried with bran, and *Glycyrrhizae Radix et Rhizome* (Gancao).

Several small-scale trials have found that XFZY is safe and effective for angina pectoris, manifested with ameliorating anginal symptoms. It relieves myocardial ischemia and has fewer adverse effects [15–17]. Pharmacology and toxicology studies based on animals have indicated the abirritation of XFZY for SA via dilating vessels, anticoagulant properties, and increasing microvessel permeability [18]. Animal studies have also demonstrated that XFZY can lower intracellular adhesion molecule-1 (ICAM-1) and vascular cell adhesion molecule-1 (VCAM-1), thus reducing inflammatory reactions triggered by ischemia-reperfusion injury (IRI) [19]. Moreover, XFZY can downregulate the expression of Forkhead box (FoxO) genes via silent information regulator 1 (SIRT 1), thus delaying ischemic heart disease [20]. A systematic review has shown that XFZY plus antianginal drugs is superior to antianginal drugs alone in mitigating symptoms, relieving myocardial ischemia, and decreasing low-density lipoprotein cholesterol (LDL-C) [21]. However, the review concluded that the included small-scale trials with poor methodological quality do not provide high-quality evidence of XFZY for the treatment of SA. Moreover, most trials in the review did not consider controlling the placebo effect that was to be identified for SA. Therefore, we designed a multicenter, double-blind, randomized placebo-controlled trial to investigate the effect and safety of XFZY for SA.

## 2. Methods

### 2.1. Objectives

This study evaluates whether XFZY as an adjunctive treatment for SA patients with *QBP* in CM produces benefits superior to those of routine care alone in alleviating angina.

### 2.2. Study Design and Setting

This study is an add-on designed, multicenter, double-blind, randomized placebo-controlled trial in which patients will be recruited from both the outpatient and inpatient cardiology departments at six centers in China. The sites include the Affiliated Hospital of Liaoning University of Traditional Chinese Medicine, the Second Affiliated Hospital of Liaoning University of Traditional Chinese Medicine, Guangdong Provincial Hospital of Chinese Medicine, First Affiliated Hospital, Heilongjiang University of Chinese Medicine, the First Hospital of China Medical University, and the Tenth People's Hospital of Shenyang. Eligible participants with SA will be randomly assigned to receive XFZY or placebo for 12 weeks and then will be followed for 12 weeks.

**Figure 1:**
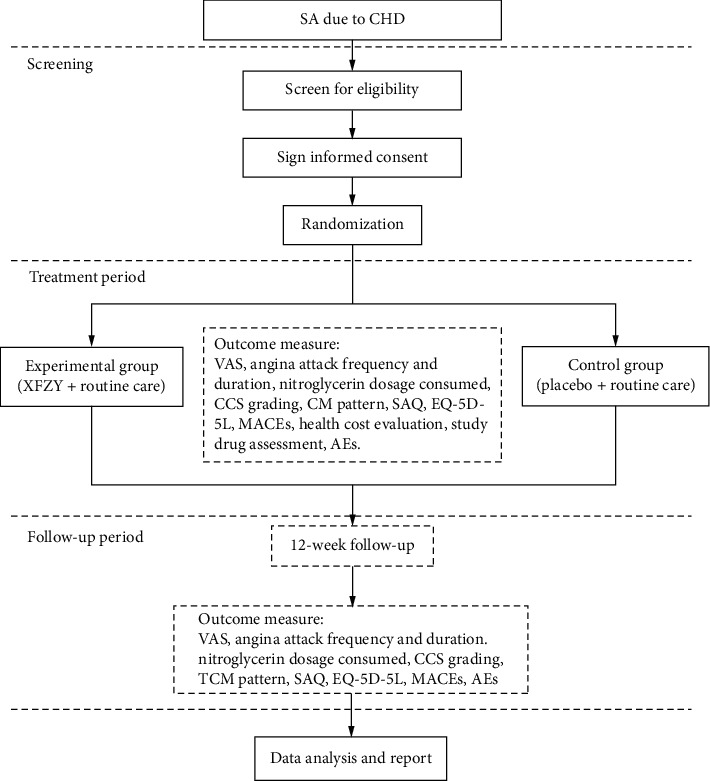
Flow diagram. SA: stable angina, CHD: coronary heart disease, VSA: visual analogue scale, CCS : Canadian Cardiovascular Society, CM : Chinese medicine, SAQ : Seattle Angina Questionnaire, EQ-5D-5 L : EuroQol-5-Dimensions-5-Level, MACEs: major adverse cardiac events, AEs: adverse events.

Figure 1 is a flowchart of the study design. The trial has been approved by the Ethics Committee at Guangdong Provincial Hospital of Chinese Medicine (No. BF2019-175-01) and registered with an identifier (ChiCTR1900026899) in the Chinese Clinical Trial Registry. Additionally, a Standard Protocol Items: Recommendations for Interventional Trials (SPIRIT) [22] figure for the schedule of enrolment, interventions, and assessments is presented in Table 1.

### 2.3. Participants

#### 2.3.1. Diagnostic Criteria for SA due to CHD

According to the *Guideline for the Diagnosis and Treatment of Patients with Stable Ischemic Heart Disease* issued by the Chinese Society of Cardiology in 2018 [23] and the *Guideline Update for the Management of Patient with Chronic Stable Angina Pectoris* released by the American Heart Association/American College of Cardiology (ACC/AHA) in 2002 [24], the diagnostic criteria for SA due to CHD are as follows:

(1) Anginal pain with the following four characteristics:
  - Location: the anginal pain usually occurs in the substernal chest; atypical locations include the epigastrium, the lower jaw or teeth, shoulder blades, arms, wrists, or fingers.
  - Property: the anginal pain generally presents with pressure, tightness or heaviness, sometimes strangling, constricting, or burning. It is occasionally accompanied by shortness of breath or other nonspecific symptoms such as fatigue or faintness, nausea, burning, restlessness, or a sense of impending doom.
  - Duration: the anginal pain usually lasts less than 10 minutes.
  - Predisposing and relieving factors: the anginal pain can be triggered by physical exertion such as walking up an incline, walking against a breeze, ingesting a heavy meal, or cold weather. It can also be triggered by emotional stress. It usually resolves within few minutes by rest or with sublingual nitrates.
(2) The anginal pain is present for at least 2 months without changes in frequency, characteristics, or triggering factors

#### 2.3.2. Grading of Angina Pectoris

*The Grading of Angina of Effort by the Canadian Cardiovascular Society* [25] will be used as a grading system to quantify SA severity (Table 2).

#### 2.3.3. Qi-Stagnation and Blood-Stasis Pattern (QBP) Diagnosis

*QBP* in CM will be diagnosed by a qualified CM physician according to the *diagnosis scale for Qi-stagnation and blood-stasis syndrome (DSQBS)* [26] (Table 3). The cut-off point for the DSQBS score is 20 points for *QBP* diagnosis.

#### 2.3.4. Inclusion Criteria

Participants who fit the following conditions will be enrolled:

(1) Meeting the diagnostic criteria of SA and *QBP* in CM
(2) Age between 30 and 75 years
(3) Males with any of the following conditions and females with *b*, *c*, *d*, or *e*:
  - Positive second quantity exercise treadmill testing
  - Positive radionuclide exercise test
  - Suffered from myocardial infarction for more than 3 months
  - Undergone percutaneous coronary intervention (PCI) for more than 12 months
  - Presentation of a stenosis ≥50% in at least one main coronary artery or its larger branches in either coronary angiography or coronary computer tomographic angiography
(4) The CCS grading of effort angina is either I, II, or III
(5) The frequency of angina attack ≥ 2 weekly and ≤6 daily
(6) Visual analogue scale (VAS) used to measure average intensity pain of angina ≥ 3 cm over the past 2 weeks
(7) Signed informed consent

#### 2.3.5. Exclusion Criteria

Participants will be excluded if they meet any of the following conditions:

1. Suffering from severe heart disease including unstable angina pectoris, severe arrhythmias (e.g., rapid atrial fibrillation, atrial flutter, paroxysmal ventricular tachycardia, Mobitz II second degree atrioventricular block, and third-degree atrioventricular block), and severe cardiopulmonary insufficiency (e.g., cardiac function grade IV and severely abnormal pulmonary function)
2. Poorly controlled hypertension (systolic blood pressure ≥ 160 mmHg or diastolic blood pressure ≥ 100 mmHg after treatment)
3. Planning to undergo coronary artery bypass grafting (CABG) or PCI during the trial
4. Suffering from any disease that triggers chest pain, such as other heart diseases, neurosis, menopausal syndrome, spondylosis disease, or gastroesophageal reflux disease (GERD)
5. Suffering from severe liver or renal dysfunction (creatinine value over the upper limit for normal reference, alanine aminotransferase, aspartate aminotransferase, or total bilirubin value over 1.5 times the upper limit for normal reference)
6. Suffering from severe primary diseases, such as diseases of the respiratory, digestive, urinary, or hemopoietic system, or tumors
7. Zung Self-rating Anxiety Scale (Zung-SAS) > 59 [27, 28] or Zung Self-rating Depression Scale (Zung-SDS) > 62 [28, 29]
8. Gravida, lactating women, or individuals planning to conceive
9. Failing to cooperate with the investigator to participate in the trial (e.g., blindness, deafness, dumbness, intellectual disability, and mental disability)
10. Participation in other clinical trials within the last 3 months
11. Having allergic constitution or being allergic to the known component of the study drug
12. Not being suitable for the trial, as judged by an investigator

### 2.4. Sample Size Calculation

The primary outcome is the change from the baseline VAS score indicating angina pain intensity to the end of the treatment at the 12^th^ week. The sample size calculation was based on Yang's study which included 96 patients treated for four weeks [30]. The study's results showed that the changes in VAS mean score for patients in the experimental group and the control group were 5.63 and 3.02, respectively. The standard deviations (SDs) of the VAS scores in the two groups were 0.44 and 0.81, respectively. We assume that the VAS score for the patients in the experimental group and the control group can drop off to 5.5 (SD: 1.5) and 3.0 (SD: 1.5), respectively. With a 5% two-tailed type I error, a power of 90%, and a superiority margin of 1.7 [31], a total of 61 patients in each group was required to detect a significant difference between the two groups. Considering a 20% dropout rate, a total of 152 participants should be included in our trial, with 76 in each group.

### 2.5. Recruitment

Patients with SA will be recruited from clinics or wards of every center through advertisements and referrals. Trained investigators from each center will identify the eligible patients based on the inclusion and exclusion criteria. SA patients will be informed of the aim, procedures, benefits, and potential hazards of the trial, as well as their rights and responsibilities for the trial. Any patient may withdraw from the study at any time without giving any reason, and neither their medical care nor their legal rights will be affected. Participants withdrawing will also be asked whether they wish to receive follow-up according to the trial schedule. Potential participants will be enrolled in the trial after knowing the purpose, benefits, risks of the trial, patient's rights, and so on and providing signed informed consent.

### 2.6. Randomization and Allocation Concealment

The randomization process will be performed by the Institute of Basic Research in Clinical Medicine (IBRCM), China Academy of Chinese Medical Sciences (Beijing, China), through a validated interactive web response system. Eligible participants will be randomly assigned to receive either XFZY or the placebo at a ratio of 1 : 1 with center-stratified and permuted block random sequence created with SAS 9.2 (SAS Institute Inc., Cary, USA). The randomization results will be concealed and maintained by IBRCM. All patients and researchers, including investigators, outcome assessors, study assistants, and statisticians involved in the trial, will be unaware of patient allocation and treatment during the trial period.

### 2.7. Blinding

This study is a double-blind trial, in which neither participants nor study personnel are able to know the group allocation or identify the treatment. The placebo will be similar to XFZY in appearance, weight, taste, packaging, and labeling. An independent team from IBRCM will supervise Jilin Aodong Yanbian Pharmaceutical Group Co. Ltd. in the labeling and packaging of the study drugs according to the randomization results. If a subject suffers from a serious adverse event (SAE) or an emergency, the study drug must be identified immediately by the investigator with the unblinding privilege via the interactive web response system. Details of the unblinding cause, date, treatment, and results will be reported in the case report form (CRF). The participants will be withdrawn from the study after unblinding.

### 2.8. Interventions

Participants enrolled in the trial will be randomly assigned to receive either routine care plus XFZY or routine care plus the placebo for 12 weeks. The XFZY and the placebo will be manufactured as oral liquids by Jilin Aodong Yanbian Pharmaceutical Co. Ltd. (Jilin, China) according to the requirements of good manufacturing practice (GMP). The placebo will consist of honey, white granulated sugar, fried brown sugar, bitters, ginseng essence, natural edible pigments, and food antiseptic. It will be similar to XFZY in appearance, weight, taste, packaging, and labeling and will be examined by the triangulation method.

#### 2.8.1. Routine Care

Routine care for SA in both groups during the trial will follow the Chinese Society of Cardiology's 2018 Guidelines for Diagnosis and Treatment of Patients with Stable Coronary Artery Disease, with medications including antiplatelet agents (e.g., aspirin and clopidogrel), beta-blockers (e.g., metoprolol), lipid-lowering agents (statins), ACE inhibitors (e.g., perindopril), nitrates (e.g., nitroglycerin), and calcium channel blockers. [19].

#### 2.8.2. Experimental Group

Participants in the experimental group will receive routine care plus XFZY oral liquid. 20 ml of XFZY will be taken orally three times per day for 12 weeks.

#### 2.8.3. Control Group

Participants in the control group will receive routine care plus 20 ml of the placebo orally three times a day for 12 weeks.

#### 2.8.4. Permitted Emergency Medication during the Trial

During the trial, patients will be permitted to take a nitroglycerin tablet (0.5 mg per tablet) sublingually in cases of unbearable angina episodes. A nitroglycerin tablet can also be taken every five minutes until the pain resolves. However, patients should be immediately transported to a hospital for further medical treatment if the angina pain does not subside after consuming three nitroglycerin tablets. Time, dosage, and frequency of taking nitroglycerin tablets will be recorded in the patient's diary and then collected in the CRF by the investigators at subsequent study visits.

#### 2.8.5. Drugs and Therapy Prohibited during the Trial

Any drugs which might alleviate angina pain or improve myocardial energy metabolism beyond routine care will be prohibited. Other prohibited drugs and therapies include any traditional Chinese medicine except for the study drugs, external therapy of CM, such as acupuncture, moxibustion, massage, or cupping.

#### 2.8.6. Monitoring Compliance

Participants will be requested to record the weekly number of oral liquids they consume in the patient diaries. Any unused oral liquids must be returned at each visit. The quantity of consumed and returned oral liquids will then be recorded in the CRF to measure compliance. Patients with compliance rates equal to or greater than 80% will be considered as having high compliance. The reasons for not taking medicine will be recorded as well.

### 2.9. Outcome Measurements

#### 2.9.1. Primary Outcome

This trial's primary outcome is the change in average angina pain intensity as measured by the VAS score from baseline to 12 weeks. The VAS can range from 0 to 10 cm, and the higher the score, the more severe the pain.

#### 2.9.2. Secondary Outcomes

The secondary outcomes are as follows: (1) angina attack frequency, (2) angina attack duration, (3) nitroglycerin dosage consumed, (4) CCS grading of effort angina, (5) CM pattern change, (6) Seattle Angina Questionnaire (SAQ) [32], (7) EuroQol-5-Dimensions-5-Level (EQ-5D-5 L) [33], (8) incidence of major adverse cardiac events (MACEs), specifically referring to rehospitalization and coronary revascularization (PCI and CABG) due to SA, (9) health cost evaluation, and (10) overall assessment of the study drugs.

### 2.10. Safety Assessment

Information on adverse events (AEs), including the occurrence time, severity, duration, adopted measures, and prognosis, must be recorded truthfully and in detail. Additionally, every AE recorded will be assessed by the investigator to analyze the causality between the AE and the studied drugs. Serious adverse events (SAEs) are defined as events which can cause hospitalization, loss of ability to work, disability, congenital deformity, or death, according to the International Council for Harmonisation of Technical Requirements for Pharmaceuticals for Human Use (ICH) guidelines [34]. Besides the abovementioned measures, in the incidence of any SAE, the principal investigator will be notified. Additionally, a report will be submitted to the ethics committee and the data and safety monitoring committee (DSMC) within 24 hours.

### 2.11. Data Collection and Management

This trial's outcome assessments will be performed at baseline, 2^nd^, 4^th^, 8^th^, 12^th^, and 24^th^ week. Data for each visit must be collected within three days before or after the scheduled time. The primary and secondary outcomes will be measured at the six time points above. Moreover, safety data will be collected by both self-reporting and laboratory tests during the trial. Participants' characteristics, such as demographic data, medical history, family history of cardiovascular disease, and stable angina drug treatment within the previous three months, will be collected at baseline. Participant compliance and concomitant medications will be monitored and recorded throughout the trial. The schedule for enrolment, interventions, assessments, and data collection is presented in Table 1.

Study data will be collected by an electronic data capture (EDC) system developed by IBRCM. Data which are collected via paper CRF will be also input into the EDC. Research personnel will be trained in standard operating procedures such as recruitment, screening, and data collection. Study monitors from an independent department of Jilin Aodong Yanbian Pharmaceutical Group Co. Ltd. will visit each site regularly, validating the quality of the data collection. The monitors will check the source documents for any inconsistent or missing data and assist research personnel with any data problems. All study documents will be classified and archived under confidential conditions.

### 2.12. Statistical Analysis

Statistical analysis will be conducted with SAS 9.2 (SAS Institute Inc., USA) in accordance with a preestablished statistical analysis plan. Data analysis for efficacy will be performed following the intention-to-treat (ITT) principle. The full analysis set will include any randomized participants who not only receive treatment but also have at least one efficacy evaluation. The per-protocol analysis set will involve eligible patients with high compliance who complete most of the treatment, visits, and outcome assessments without serious protocol violations. The safety analysis set will include all postrandomization patients who receive at least one treatment to assess the safety of the study drug. Missing data will be addressed with the multiple imputation method.

For the primary and secondary outcomes, *t*-test or Mann–Whitney *U* test will be employed for continuous variables with normal or unknown distributions; Chi-squared test or Fisher's exact test will be used for categorical variables. Change across time among the primary and secondary outcomes will be analyzed by repeated measures of analysis of variance or a linear mixed model. The two groups' baseline characteristics will be summarized by descriptive statistics.

Factors such as center, sex, age, course of the disease, and VAS score at baseline will be considered covariates to be adjusted in the statistical model. For safety assessments, a safety analysis set will be adopted to detect whether the incidence of AEs is significant between groups. This will be done with either a chi-squared test or Fisher's exact test. All AEs will be coded according to the Chinese version of the Medical Dictionary for Regulatory Activities (MedDRA). Findings will be considered statistically significant if the two-sided alpha level is less than 0.05.

## 3. Discussion

Although there are numerous effective pharmacological treatments and coronary revascularizations, SA remains a significant cause of poor quality of life and even disability for many patients with ischemic heart disease. Many patients declare that adequate symptom control is difficult to achieve. In China, most SA patients and physicians seek help from CM, pointing out that XFZY, as a Chinese herbal patent product and conjunctive therapy, can alleviate angina pain. Using an add-on design, this multicenter, double-blind, randomized placebo-controlled trial can investigate whether XFZY is more effective for SA than routine care alone.

In addition to the small-scale trials' results supporting XFZY for SA [15–17], preclinical research may also provide a partial explanation for the trial's rationale. The results of animal and cytologic studies have revealed that XFZY inhibits platelet aggregation by blocking the activity of glycoprotein lIb/IIIa complex on the surface of the platelet membrane, relieving ischemia-reperfusion injury to the myocardium by downregulating lipid peroxidation, and promoting angiogenesis in the ischemic region by inducing endothelial progenitor cell migration [35–37].

Regardless, several potential limitations of this study should be considered, such as the VAS score. This is the average intensity over the past two or four weeks of angina pain as shown by the VAS score at each visit that patients self-report. Additionally, relying on recall might not accurately reveal pain intensity in prevailing circumstances. This is another limitation of this trial. However, the pain severity due to stable angina typically remains stable for several months. Due to the original intention of this study, it was not possible to conduct a subgroup analysis of the efficacy of XFZY for patients with stable angina post-PCI or post-CAGB. In view of inadequate sensitivity and specificity [38], triggering angina resulting in patients' noncompliance, and strict and time-consuming procedures leading to doctors' violation protocol, failing to consider treadmill exercise test to evaluate control of ischemia or effort performance is also a limitation.

In conclusion, this trial will provide high-quality evidence for XFZY for SA as a conjunctive therapy. Whether the results are neutral, negative, or positive, this trial will have clinical implications for patients with SA.

## Figures and Tables

**Table 1:** Content for the schedule of enrolment, interventions, and assessments.

Study phase	Run-in period	Treatment period					Follow-up period
Time	Visit 1	Baseline	Visit 1	Visit 2	Visit 3	Visit 4	Visit 5
	Week 2	Week 0	Week 2	Week 4	Week 8	Week 12	Week 24
Patient							
Inclusion/exclusion criteria	×						
Informed consent	×						
Random grouping	×						
Demographic data	×	×					
Allergic history	×						
Personal history	×						
Previous history	×						
Family history of cardiovascular disease	×						
Drug treatment for SA within the past three months	×						
Interventions							
Experimental group		×	×	×	×	×	
Control group		×	×	×	×	×	
Outcomes measurements							
Average angina pain intensity (VAS)	×	×	×	×	×	×	×
Angina attack frequency	×	×	×	×	×	×	×
Angina attack duration		×	×	×	×	×	×
Nitroglycerin dosage		×	×	×	×	×	×
CCS grading of effort angina	×	×	×	×	×	×	×
Change of CM pattern		×	×	×	×	×	×
SAQ		×		×	×	×	×
EQ-5d-5 L		×	×	×	×	×	×
MACEs		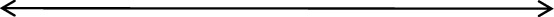					
Health cost evaluation		×	×	×	×	×	
Overall drug assessment						×	
Safety assessment							
Vital signs	×	×	×	×	×	×	×
Routine blood and urine analysis, liver and renal function tests, coagulation function test, ECG and UCG		×				×	
Adverse events		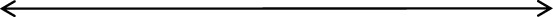					
Other work							
Concomitant medications		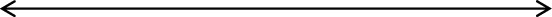					
Compliance measurement		×	×	×	×	×	

SA: stable angina, VSA: visual analogue scale, CCS : Canadian Cardiovascular Society, CM : Chinese medicine, SAQ : Seattle Angina Questionnaire, EQ-5D-5 L : EuroQol-5-Dimensions-5-Level, MACEs: major adverse cardiac events, ECG : Electrocardiogram.

**Table 2:** Grading of angina of effort by the Canadian Cardiovascular Society.

I	Ordinary activity, such as walking and ascending stairs, does not cause angina; angina with strenuous or rapid or prolonged exertion at work or recreation
II	Slight limitation of ordinary activity; angina walking or ascending stairs rapidly, walking or ascending stairs after meals, or in the cold, wind or under emotional stress, or only during the first few hours awake, walking more than 200 m on level ground or ascending more than one flight of ordinary stairs at a normal pace under normal conditions
III	Marked limitations in ordinary physical activity; angina when walking 100–200 m on level ground or one flight of stairs under normal conditions and at a normal pace
IV	Inability to partake in any physical activity without discomfort—angina syndrome may be present at rest

**Table 3:** Diagnosis scale for Qi-stagnation and blood-stasis syndrome.

Symptoms and signs	Yes	No	Score
Pain	9	0	______
Irritability/depression	16	0	______
Distending pain	2	0	______
Scurry pain	6	0	______
Chest distress	0.5	0	______
Lumps in body	7	0	______
Petechia in the tongue	4	0	______
Purplish tongue	1	0	______
Uneven pulse	4	0	______
Deep pulse	2	0	______
If the sum ≥20, *QBP* can be diagnosed.		Score:	______

*Notes*. Pain includes stomachache, abdominal pain, low back pain, dysmenorrhea, breast pain, and limb pain. QBP: qi-stagnation and blood-stasis pattern.

## Data Availability

Data sharing is not applicable to this article as no databases were generated or analyzed during the current stage. However, data are available from the corresponding author upon reasonable request after the study is complete.

## Abbreviations

SA: Stable angina
XFZY: Xuefu Zhuyu formula
RCT: Randomized controlled trial
CHD: Coronary heart disease
CM: Chinese medicine
QBP: qi-stagnation and blood-stasis pattern
IRI: Ischemia-reperfusion injury
CCS: Canadian Cardiovascular Society
PCI: Percutaneous coronary intervention
VAS: Visual analogue scale
CABG: Coronary artery bypass grafting
SD: Standard deviation
IBRCM: Institute of Basic Research in Clinical Medicine
AE: Adverse event
CRF: Case report form
SAQ: Seattle angina questionnaire
EQ-5D-5 L: EuroQol-5-dimensions-5-level
MACE: Major adverse cardiac event
EDC: Electronic data capture
ITT: Intention-to-treat.

## Additional Points

The trial is currently recruiting patients.

## Supplementary Materials

Supplementary Materials SPIRIT 2013 checklist: recommended items to address in a clinical trial protocol and related documents.
